# Role of Oxidative Stress in Transformation Induced by Metal Mixture

**DOI:** 10.1155/2011/935160

**Published:** 2011-12-04

**Authors:** Silva-Aguilar Martín, Rojas Emilio, Valverde Mahara

**Affiliations:** Departamento de Medicina Genómica y Toxicología Ambiental, Instituto de Investigaciones Biomédicas, Universidad Nacional Autónoma de México, Ciudad Universitaria 04510, DF, Mexico

## Abstract

Metals are ubiquitous pollutants present as mixtures. In particular, mixture of arsenic-cadmium-lead is among the leading toxic agents detected in the environment. These metals have carcinogenic and cell-transforming potential. In this study, we used a two step cell transformation model, to determine the role of oxidative stress in transformation induced by a mixture of arsenic-cadmium-lead. Oxidative damage and antioxidant response were determined. Metal mixture treatment induces the increase of damage markers and the antioxidant response. Loss of cell viability and increased transforming potential were observed during the promotion phase. This finding correlated significantly with generation of reactive oxygen species. Cotreatment with *N*-acetyl-cysteine induces effect on the transforming capacity; while a diminution was found in initiation, in promotion phase a total block of the transforming capacity was observed. Our results suggest that oxidative stress generated by metal mixture plays an important role only in promotion phase promoting transforming capacity.

## 1. Introduction

Arsenic (As), cadmium (Cd), and lead (Pb) are commonly used in numerous industries to the extent that they have now generated a pollution problem. Numerous studies have reported high levels of these metals near smelter areas [1]. Acute exposure to As, Cd, and Pb produces a variety of toxic effects in several target organ systems; however, most individuals are chronically exposed to low levels of a mixture of these metals [2, 3]. These three metals/metalloids share several common mechanisms underlying their toxicities, including production of oxidative stress, reaction with sulfhydryl groups, and interference with essential metals. In addition, stress proteins and antioxidant enzymes have been proposed to provide common cellular protective mechanisms against the element-induced toxicities when they occur on an individual basis [4]. Furthermore, these metals have been listed in the top ten hazardous substances and proposed as one of the mixtures for interaction profile studies by the Agency for Toxic Substances and Disease Registry (ATSDR). As and Cd have been classified as carcinogens and Pb as a possible carcinogen by the International Agency in Research of Cancer (IARC) [5–9]. 

As, Cd, and Pb induce the generation of reactive oxygen species (ROS), which can damage DNA, lipids, and proteins. As generates ROS in the form of superoxide (O2^•−^), singlet oxygen (_1_O^2^), peroxyl radical (ROO^•^), nitric oxide (NO^•^), hydrogen peroxide (H_2_O_2_), dimethylarsinic peroxyl radicals ([(CH_3_)_2_AsOO^•^]), and the dimethylarsinic radical [(CH_3_)_2_As^•^] [10, 11]. Cd generates ROS in the form of superoxide (O2^•−^), hydrogen peroxide (H_2_O_2_), hydroxyl radical (HO^•^), and lipid radicals (L^•^). In addition, Cd treatment can cause the replacement by iron in some enzymes, and the accumulated iron molecules reacts with H_2_O_2_ to produce hydroxyl radicals (HO^•^) [10, 11]. Pb ROS-generating mechanism is mediated by delta-aminolevulinic acid dehydratase (*δ*-ALAD) inhibition, which provokes the accumulation of delta-aminolevulinic acid (*δ*-ALA). *δ*-ALA is rapidly oxidized to generate free radicals such as superoxide (O2^•−^), hydroxyl radicals (HO^•^), and hydrogen peroxide (H_2_O_2_) [10, 12].

Interactions between As, Cd, and Pb enhance the risk of cancer in certain human populations. However, the carcinogenic mechanisms associated with these metal mixtures have been poorly studied. Numerous possible mechanisms of action have been proposed: oxidative stress inductions, genotoxicity, DNA repair inhibition, and gene expression changes [13]. Among these proposed mechanisms, oxidative stress is commonly observed in *in vivo* and *in vitro *systems during exposure to these metals as mixture [4]. However, several studies suggest that certain metal-metal interactions inhibit carcinogenic activity [14]. Because humans are continually exposed to complex metal mixtures at low doses, it is necessary to determine the mechanism underlying the transformation process.

Cell transformation is a hallmark of carcinogenic activity [15]. There are two general categories of transformation systems based on the target cells used for the assay: diploid cells with limited *in vitro* life span (such as Syrian Hamster Embryos) and immortalized aneuploid cell lines (such as BALB/c 3T3 and C3H/10T1/2 cells) [16]. The most commonly employed target cells include BALB/c 3T3 A31-1-1 cells. The initiation and promotion of carcinogenesis are commonly studied in BALB/c 3T3 A31-1-1 cells [16–19]. Individually, the transformation capacity of As, Cd, and Pb has been tested previously in these cells; both As and Cd give a positive response, whereas Pb does not display transformation [16, 20–22]. The aim of this work was to evaluate the transforming potential of the metal mixture (2 *μ*M NaAsO_2_ + 2 *μ*M CdCl_2_ + 5 *μ*M Pb(C_2_H_3_O_2_)_2_·3H_2_O) at relevant epidemiological concentrations, similar to those found in occupational exposure individuals [23–25], because has not been tested in this model.

In the present study we evaluated the role of reactive oxygen species and the antioxidant barrier in the development of oxidative stress damage through transforming processes. This process was examined during the initiation and promotion phases of the two-step transformation model in BALB/c 3T3 A31-1-1 cells. The transforming potential of the metals mixture as initiator and/or promoter was also determined. We found that oxidative stress plays an important role in transformation induced by the metal mixture and is most prominent during the promotion phase of cell transformation.

## 2. Material and Methods

### 2.1. Chemicals

Sodium meta-arsenite (NaAsO_2_, purity 100%), cadmium chloride (CdCl_2_, purity 99.5%), 12-O-tetradecanoylphorbol-13-acetate (TPA), n-methyl-n-nitrosoguanidine (MNNG), 6-hydroxy-2,5,7,8-tetramethylchromane-2-carboxylic acid (Trolox), rhodamine 123 and dihydrorhodamine 123, were purchased from the Aldrich Chemical Co. (Milwaukee, Wis/USA). Lead acetate (Pb(C_2_H_3_O_2_)_2_·3H_2_O, purity 99.9%), insulin-transferrin-selenium-A (ITS-A), and 1,1,3,3-tetraethoxypropane were purchased from J. T. Baker (México), GIBCO/Invitrogen (NY, USA) and Fluka Chemie Co. (USA), respectively.

### 2.2. Cell Culture

The morphological transformation experiments were performed using BALB/3T3 A31-1-1 clonal cells (ATCC). Cells were grown in Dulbecco's modified Eagle's minimum essential medium (DMEM) supplemented with 10% FBS in a humidified incubator under 95% air and 5% CO_2_. Cells were subcultured before reaching confluence, usually twice per week. Additional media used during the promotion stage of the transformation assay consisted of the following: Dulbecco's modified Eagle's minimum essential medium (DMEM) supplemented with 2% FBS and 1% ITS-A (10 mg/mL insulin, 5.5 mg/mL transferring, and 0.0067 mg/mL sodium selenite). All media were obtained from the GIBCO/Invitrogen (NY, USA).

### 2.3. Transformation Assay

**Figure 1:**
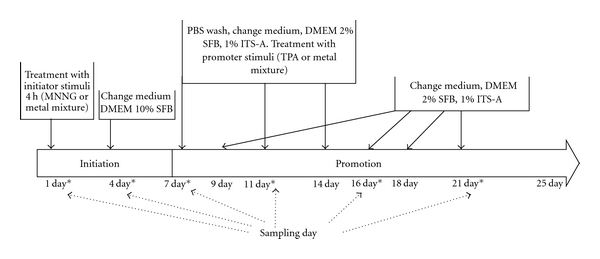
Scheme of two-phase transformation protocol, Balb/c 3T3 cells. Initiation phase, day 1 to 7. On day 1, subconfluent cell culture was treated with MNNG 0.5 *μ*g/mL (positive initiator) or metal mixture (2 *μ*M NaAsO_2_, 2 *μ*M CdCl_2_, and 5 *μ*M Pb(C_2_H_3_O_2_)_2_·3H_2_O) as initiator stimuli during 4 h and reseeded in DMEM medium supplemented with 10% SFB. On day 4 medium was changed. Promotion phase begins on day 7 and ends on day 25. In promotion phase, cells were cultured in DMEM medium supplemented with 2% SFB and 1% ITS-A. On days 7, 11, and 14 cells were treated with TPA 0.1 *μ*g/mL (positive promoter) or metal mixture (2 *μ*M NaAsO_2_, 2 *μ*M CdCl_2_ and 5 *μ*M Pb(C_2_H_3_O_2_)_2_·3H_2_O) as promoter stimuli. Meanwhile, on days 9, 16, 18, and 21 medium changes were done. Sampling days across transformation protocol are represented by “*”; in these days samples were taken before changing media or applying treatment.

The transformation assay was performed as described previously with slight modifications [22]. The transformation protocol consisted in 25 days, divided into two phases: initiation phase between days 1 to 7 and promotion between days 7 to 25. BALB/c 3T3 A31 cells were plated at a density of 5 × 10^5^ cells per 100 mm dish in DMEM medium supplemented with 10% FBS. After 48 h incubation on day 1, subconfluent cells were exposed to the metal mixture (2 *μ*M NaAsO_2_, 2 *μ*M CdCl_2_, and 5 *μ*M Pb(C_2_H_3_O_2_)_2_·3H_2_O) or MNNG (0.5 *μ*g/mL), as initiators. The media were removed 4 hours later, and the cells for initiation screening were replated at a density of 1 × 10^3^ cells per 60-mm dish. On day 4, the media were replenished for all treatments and controls. On day 7, the dishes were replenished with medium supplemented with 1% ITS-A and 2% FBS. The metal mixture or TPA (0.1 *μ*g/mL) was then added as promoters. These media and treatments with metal mixture or TPA were replenished on days 11 and 14. In days 9, 16, 18, and 21, the cells were reseeded only in fresh media DMEM + 1% ITS-A + 2% FBS. On day 25, the cells were fixed with ethanol and stained with the Giemsa solution (see Figure 1). To examine the initiating effects of metals, cells were exposed to the metal mixture during the initiation stage and then treated with TPA during the promotion stage. To determine promotional effect, cells were treated with MNNG as initiator and exposed to the metal mixture during the promotion stage. In addition, the effects of metal mixture were examined as initiator and promoter stimuli. Treatments with MNNG as an initiator and TPA as a promoter were used as positive controls for cell transformation [18]; also, both were tested individually. The study groups will be mentioned as initiator/promoter. Samples used for analysis of the initiation phase were collected on day 1 after 4 hours of treatment and on days 4 and day 7 before the promoter treatment was started (Figure 1). Promotion samples were obtained on days 11, 16, and 21 (Figure 1). Transformed foci type III were scored according to the following criteria, which discriminate these foci based on four morphological characteristics: (1) foci of more than 2 mm in diameter, (2) deep basophilic staining, (3) dense multilayering of cells, and (4) random orientation of cells at the edge of the foci [16, 18, 21, 22]. Data were analyzed using an optimized model as described by Ponti et al. [26]. To evaluate the transforming potential (TP), we count the number of transformed foci type III per dish obtained for each experimental condition and adjust it with respect to the number of surviving cells at each sampling day.

### 2.4. Viability

Cell viability was measured by the dual stain fluorescein diactetate (FDA) method as described by Rojas et al. [27]. Briefly, the cells were mixed with a fluorochrome solution containing 0.02 *μ*g/mL ethidium bromide and 0.015 *μ*g/mL FDA. Cells were then analyzed under a fluorescence microscope (Olympus BMX-60 with a UM61002 filter); green-stained cells were identified as live, while red-stained cells were identified as death cells. One hundred randomly chosen cells were evaluated per condition, and the results are expressed as percentages.

### 2.5. Reactive Oxygen Species (ROS)

The dihydrorhodamine 123 technique is based on the reactive-oxygen-species- (ROS-) dependent oxidation of dihydrorhodamine-123 to rhodamine-123 [28]. Briefly, 100 *μ*L aliquots of the harvested samples were collected and centrifuged at 1200 rpm for 5 minutes. The supernatant was then discarded, and 180 *μ*L of buffer A (140 mM NaCl, 5 mM KCl, 0.8 mM MgSO_4_ 7HÒ, 1,8 mM CaCl_2_, 5 mM glucose and 15 mM HEPES) and 20 *μ*L of dihydrorhodamine 123 (1 *μ*M) were added. The absorbance of the rhodamine 123 formed was measured at 505 nm using an ELISA spectrophotometer (Bio-Rad Model 550) and interpolated in a curve of rhodamine 123 in concentrations of 0–10 *μ*M.

### 2.6. Lipid Peroxidation (LPx)

The thiobarbituric acid method was used to measure the concentration of malondialdehyde (MDA) [29]. A 100 *μ*L aliquot was added to 100 *μ*L of trichloroacetic acid (10% w/v) and centrifuged at 3000 ×g for 10 minutes. The supernatant was then added to 1 mL of the thiobarbituric acid reagent (0.375%), and the mixture was heated at 92°C for 45 minutes. The absorbance of the thiobarbituric acid-MDA complex was measured at 532 nm using an ELISA spectrophotometer (Bio-Rad Model 550). Data were interpolated onto a concentration curve of 1,1,3,3-tetraethoxypropane ranging from 0 to 10 nM.

### 2.7. Genotoxicity. Single-Cell Gel Electrophoresis (SCGE) Assay

Ten microliters of the cell suspension (10,000–15,000 cells) was mixed with 75 *μ*L of a 0.5% LMP agarose solution (0.36% final) and loaded onto microscope slides prelayered with 150 *μ*L of 0.5% normal melting point agarose. The SCGE assay was performed as described by Vega et al. [30]. Briefly, after incubation with lysis buffer (2.5 M NaCl, 100 mM EDTA, 10 mM Tris, pH 10, supplemented with 10% DMSO and 1% Triton X-100) at 4°C for at least 1 h, the slides were placed in a horizontal electrophoresis chamber containing running buffer solution (300 mM NaOH, 1 mM EDTA, pH > 13). The slides remained in the electrophoresis buffer for 10 min to allow the DNA to unwind. Electrophoresis was performed for 10 min at 300 mA and 25 V (~0.8 V/cm), and all technical steps were conducted using very dim indirect light. After electrophoresis, the slides were gently removed and rinsed with neutralization buffer (0.4 M Tris, pH 7.5) at room temperature for 15 min. The slides were dehydrated with 100% ethanol (15 min), after which they were air-dried. Ethidium bromide (20 *μ*L of a 0.2 *μ*g/mL solution) was added to each slide, and a coverslip was placed on the gel. Individual cells were visualized at 20x magnification using an Olympus BX-60 microscope with fluorescence attachments (515–560 nm excitation filter, 590 nm barrier filter). Images were digitized and analyzed using KOMET v.31 software (Kinetic Imaging), and the Olive tail moment (OTM) parameter was used to evaluate DNA damage (200 cells were scored for each condition).

### 2.8. Determination of Catalase Activity

Catalase activity was measured as described by Aebi [31]. Briefly, the cells were washed two or three times with sterile PBS containing protease inhibitors. The cells were sonicated for 10 cycles of 10 seconds each at 20 MHz. After sonication, the cells were centrifuged at 10,000 rpm for 5 minutes at 4°C. Catalase activity and the protein concentration were measured in the supernatant. To measure catalase activity, the absorbance of 100 *μ*L of supernatant was determined at 240 nm in phosphate buffer (50 mM) at room temperature. After the addition of 20 mM H_2_O_2_, the absorbance was recorded every 15 seconds over a period of 1 minute. Data analysis was performed as described by Aebi [31].

### 2.9. Determination of Superoxide Dismutase (SOD) Activity

Superoxide dismutase activity was measured following the protocol proposed by Sun et al. [32]. This method is based on the competition between superoxide dismutase and tetrazolium blue for the superoxide radicals formed from the xanthine oxidase reaction. Cells were sonicated and centrifuged at 10,000 rpm for 10 minutes at 4°C. Next, 200 *μ*L of the supernatant was divided into two tubes containing 1.85 mL of the reaction mix (0.265 mM xanthine, 0.53 mM EDTA, 0.1325 mM NBT, 883 mg/mL albumin, 353 mM Na_2_CO_3_). Fifty microliters of 50 mM phosphate buffer (blank) was added to one tube, and 50 *μ*L of xanthine oxidase (2–2.5 U/mL) was added to the other tube. The tubes were incubated for 15 minutes, and then 500 *μ*L of a CuCl_2_ solution was added to stop the reaction. Two hundred microliters of the reaction mixture was added to a 96-well microplate, and the absorbance was measured at 560 nm. The units of SOD were calculated as follows:

(1) $$\text{units}\;\text{of}\;\text{SOD}=\frac{\left[{\text{A}}_{\text{reaction}\;\text{mix}}-\left({\text{A}}_{\text{sample}}-{\text{A}}_{\text{blank}}\right)\right]}{\left[{\text{A}}_{\text{reaction}\;\text{mix}}\;\left(0.5\text{\%}\right)\right]}.$$

A unit of SOD is defined as the quantity of enzyme required to decrease the absorbance by 50%.

### 2.10. Total Antioxidant Capacity (TAC)

The total antioxidant capacity was measured as described by Erel [33] with modifications. This method is based on the reduction of the ABTS^•+^ radical. ABTS^•+^ is generated by an initial incubation with hydrogen peroxide in an acidic medium (final concentration of ABTS^•+^: 10 mM). Next, 5 *μ*L of the sample was mixed with 200 *μ*L of reagent 1 (0.4 M acetate buffer; ph 5.8) in a 96-well plate. Twenty microliters of reagent 2 (30 mM ABTS^•+^  in acetate buffer; pH 3.6) was then added to the mixture, and the absorbance was read before mixing R1 with R2 (blank). One final absorbance reading was obtained at the end of the 5 min incubation period at 740 nm. The reaction rate was calibrated and interpolated on a curve of Trolox absorbance (0–100 *μ*M). The TAC measurement assay results were obtained as the Trolox equivalent/L. 

## 3. Results

### 3.1. *In Vitro* Cell Transformation

The transforming potential of the metal mixture (2 *μ*M NaAsO_2_, 2 *μ*M CdCl_2_ and 5 *μ*M Pb(C_2_H_3_O_2_)_2_·3H_2_O) was determined based on an endpoint test which uses the number of *foci* per plate stained at day 25 of the assay, adjusted with the number of surviving cells at every sampling day per condition. Under this analysis we present transforming potential (TP) data across initiation (Table 1) and promotion phase (Table 2) for all experimental conditions. The metal mixture demonstrated a high degree of transforming potential during both the initiation and the promotion phases of transformation. Punctually in Table 1, metal mixture as initiator stimuli is the unique experimental condition that clearly shows positive TP in addition to positive control, MNNG/TPA (initiator/promoter), while metal mixture as promoter and metals/metals show TP statistically significant at day 7. However through promotion phase (Table 2), TP was positive for all experimental conditions being greatest at day 11 of the transforming process. Across promotion phase, metal mixture as initiator treatment decreases TP, while metal mixture as promoter or both initiator and promoter increases TP, showing an additive behavior. Furthermore, the effects of metal mixture were higher than those observed for the positive controls (MNNG/TPA); also, neither MNNG nor TPA showed transforming capacity (data not shown).

### 3.2. Effects of the Metal Mixture on the Initiation Phase

**Figure 2:**
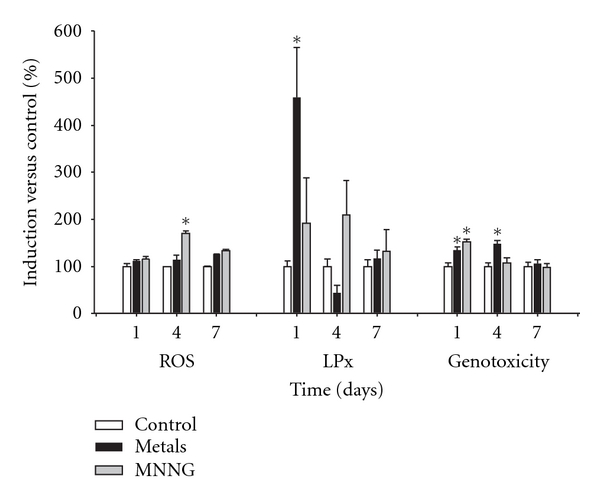
Effects of the metal mixture through initiation phase of transformation process, oxidative damage markers. Balb/c 3T3 cells were exposed to an initiator stimuli, metal mixture (2 *μ*M NaAsO_2_, 2 *μ*M CdCl_2_, and 5 *μ*M Pb(C_2_H_3_O_2_)_2_·3H_2_O) or MNNG (positive initiator), on day 1. Samplings were on days 1, after 4 hours of initiator exposure, and on days 4 and 7 before changing medium. Data represent the mean of 3 individual experiments performed by triplicate. We evaluated the generation of reactive oxygen species (ROS) using dihydrorhodamine-123 oxidation. Lipid peroxidation (LPx) was assessed using the thiobarbituric acid method, and genotoxicity was determined using the alkaline comet assay. ANOVA and Student's *t*-test; *P < 0.05 versus control bars.

**Figure 3:**
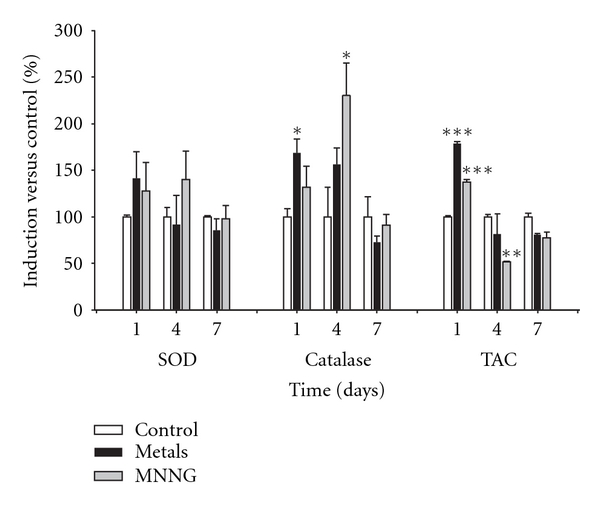
Effects of the metal mixture through initiation phase of transformation process, antioxidant activity markers. Balb/c 3T3 cells were exposed to initiator stimuli, metal mixture (2 *μ*M NaAsO_2_, 2 *μ*M CdCl_2_, and 5 *μ*M Pb(C_2_H_3_O_2_)_2_·3H_2_O) or MNNG (positive initiator), on day 1. Samplings were on days 1, after 4 hours of initiator exposure, and on days 4 and 7 before changing medium. Data represent the mean of 3 individual experiments performed by triplicate. Antioxidant activity is represented as a percentage with respect to control values. Superoxide dismutase (SOD) and catalase activities were evaluated using spectrophotometric assays, and the total antioxidant capacity (TAC) was assessed by the ABTS° + radical method (ANOVA and Student's *t*-test; *P < 0.05, **P < 0.001, ***P < 0.0001 versus control bars).

Chemical carcinogenesis is a multistep process that involves morphological cell transformation, which measures the carcinogenic potential during both the initiation and the promotion phases [20, 34]. We determined the mechanism by which the metal mixture affects oxidative stress during the initiation phase using samples from days 1, 4, and 7 (Figures 2 and 3).

ROS generation was positive in day 4 of samples treated with MNNG. Lipid peroxidation and genotoxicity were observed after treatment with the metal mixture for four hours on day 1. Genotoxicity was also induced on day 4, but neither lipid peroxidation nor genotoxicity was observed on day 7 (Figure 2).

After treatment with the metal mixture, catalase and total antioxidant capacity activation were detected on day 1 (Figure 3). These results indicate that the cells had undergone transformation with a concomitant decrease in viability (Table 1). Treatment-induced oxidative stress was suppressed by the cellular antioxidant response.

### 3.3. Effects of the Metal Mixture on the Promotion Phase of Transformation

**Figure 4:**
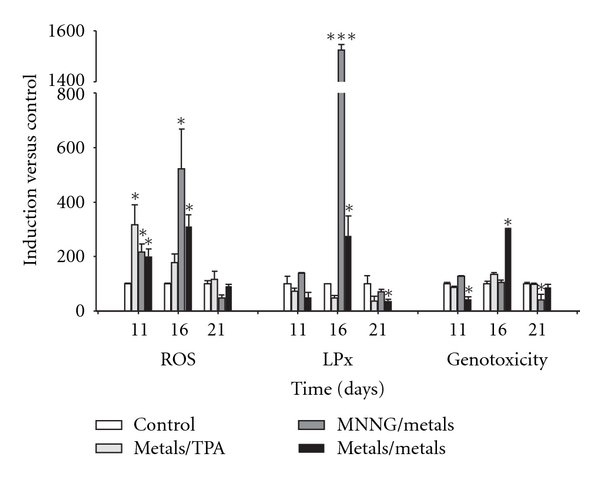
Effects of the metal mixture (2 *μ*M NaAsO_2_, 2 *μ*M CdCl_2_, and 5 *μ*M Pb(C_2_H_3_O_2_)_2_·3H_2_O) through promotion phase of transformation process, oxidative damage markers. Balb/c 3T3 cells were exposed 4 hours to initiator stimuli, metal mixture or MNNG (positive initiator), on day 1 and promoter stimuli, metal mixture or TPA (positive promoter), on days 7, 11, and 14. Samplings were on days 11, 16, and 21 of the transformation protocol for monitoring promotion phase. Data represent the mean of 3 individual experiments performed by triplicate. We evaluated the generation of reactive oxygen species (ROS) using dihydrorhodamine-123 oxidation. Lipid peroxidation (LPx) was assessed using the thiobarbituric acid method, and genotoxicity was determined using the alkaline comet assay. ANOVA and Student's *t*-test; *P < 0.05, ***P < 0.0001 versus control bars.

**Figure 5:**
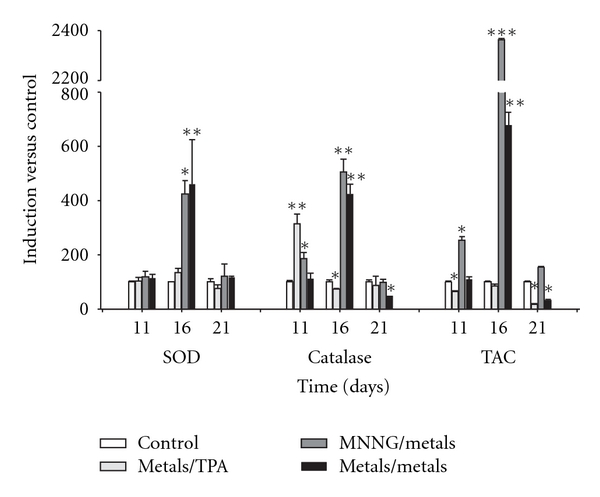
Effects of the metal mixture (2 *μ*M NaAsO_2_, 2 *μ*M CdCl_2_, and 5 *μ*M Pb(C_2_H_3_O_2_)_2_·3H_2_O) through promotion phase of transformation process, antioxidant activity markers. Balb/c 3T3 cells were exposed 4 hours to initiator stimuli, metal mixture or MNNG (positive initiator), on day 1 and promoter stimuli, metal mixture or TPA (positive promoter), on days 7, 11, and 14. Samplings were on days 11, 16, and 21 of the transformation protocol for monitoring promotion phase. Data represent the mean of 3 individual experiments performed by triplicate. Antioxidant activity is represented as a percentage with respect to control values. Superoxide dismutase (SOD) and catalase activities were evaluated using spectrophotometric assays, and the total antioxidant capacity (TAC) was assessed by the ABTS° + radical method (ANOVA and Student's *t*-test; *P < 0.05, **P < 0.001, ***P < 0.0001 versus control bars).

The promotion phase of cellular transformation occurs after chronic exposure to a promoter agent. BALB/C 3T3 cells can be used as a model of this effect in the transformation protocol [35]. The effects of the metal mixture on oxidative stress during this stage were measured on days 11, 16, and 21 (Figures 4 and 5). 

In all treatments, we observed a dramatic decrease in cell viability on day 11 (Table 2). However, on day 16, just the cells treated with the metal mixture/TPA (initiator/promoter) demonstrated recovery to control levels. On day 11, the transforming potential determined for every treatment was similar to that induced by the positive control. However, this effect decreased over time. On day 21, the metal mixture promoter treatment induced transformation to a level similar to the TPA promoter treatment (Table 2). 

Oxidative stress was determined based on the levels of measured reactive oxygen species, lipid peroxidation, and genotoxicity. These parameters were highly increased after treatment with the metal mixture during the promotion phase on day 16. However, only ROS generation was detected on day 11 for every treatment (Figure 4). 

Antioxidant activity was determined by measuring catalase and SOD activities, as well as TAC. These activities were highly increased on day 16, but only catalase activity was increased on day 11 after treatment with the metal mixture/TPA and MNNG/metal mixture (Figure 5). During the promotion phase, we observed a large elevation in oxidative stress markers (day 16), resulting in a decrease in cell viability at day 21 (Table 2). Those results suggest a cloning selection process for cells that have the capacity to resist oxidative stress.

### 3.4. Pearson Correlation Analysis and Multivariate Analysis

To determine the influence of oxidative stress on the transformation process, we performed a statistical analysis to evaluate the effects of treatment with the metal mixture during both the initiation and the promotion phases.  Table 3 shows the relationship between oxidative markers and antioxidant response markers; however, only ROS and cell viability correlated with the transforming potential. We proceeded to study this relationship using multivariate analysis with multiple linear regression. A model was obtained for each variable; however, the transforming potential was predicted only by cell viability and ROS induction (Table 4). These results suggest that ROS influences transformation and viability via the generation of oxidative stress and challenging antioxidant response.

### 3.5. Influence of NAC on Transformation Induced by the Metal Mixture; Determination of Cell Viability, Transforming Capacity, and Lipid Peroxidation

*N*-Acetyl-cysteine (NAC) is a cysteine donor that promotes the reduction of glutathione (GSH). NAC acts as an antioxidant in chelation therapy for metal detoxification. Based on these characteristics, we performed a metals-NAC cotreatment in an attempt to block the metal-mixture-induced oxidative stress and transformation [36]. We coexposed the cells with 10 mM NAC and metal mixture (2 *μ*M NaAsO_2_, 2 *μ*M CdCl_2_ and 5 *μ*M Pb(C_2_H_3_O_2_)_2_·3H_2_O) across transformation protocol. 

**Figure 6:**
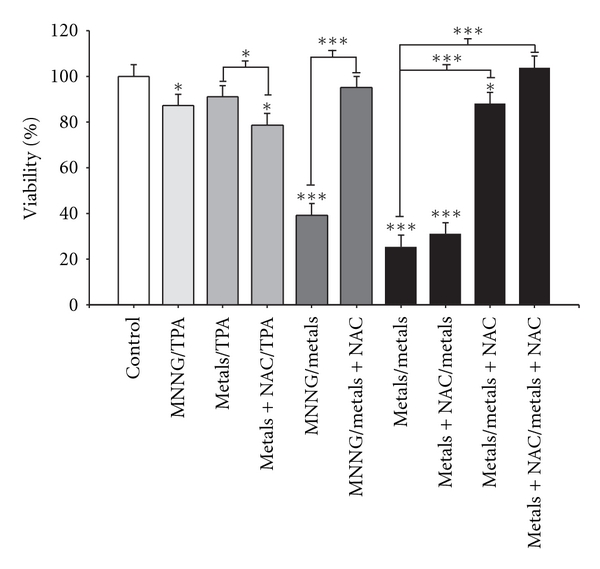
Influence of *N*-acetyl-cysteine (NAC) on metal-mixture-induced transformation, cell viability determination. Cells were cotreated with 10 mM NAC and the metal mixture (2 *μ*M NaAsO_2_, 2 *μ*M CdCl_2_, and 5 *μ*M Pb(C_2_H_3_O_2_)_2_·3H_2_O); samples were collected on day 16. Data represent the mean of three independent experiments performed in triplicate. Cell viability is presented as the percentage with respect to control values, as determined by the metabolic dual stain. ANOVA and Student's *t*-test *P < 0.05, ***P < 0.0001.

We determined the effects of NAC on cell viability and lipidperoxidation on day 16 of the protocol because this time point elicited the greatest induction of oxidative stress. A loss of cell viability was observed following NAC cotreatment as initiator stimuli and TPA as promoter treatment. However, viability recovery was observed in NAC cotreatment as promoter stimuli; these effects were similar to those determined for the controls (Figure 6). 

NAC cotreatment, as initiator stimuli, did not affect lipid peroxidation; however, these treatments as promoter stimuli abolished metal-mixture-induced lipid peroxidation during the promotion phase (Figure 8). 

**Figure 7:**
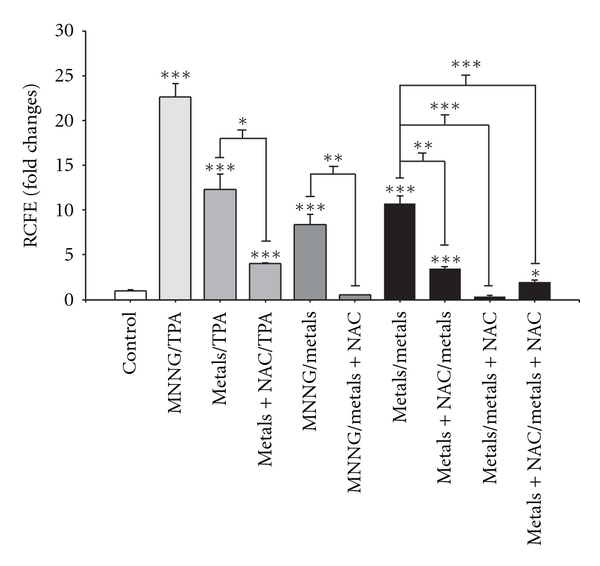
Influence of *N*-acetyl-cysteine (NAC) on metal-mixture-induced transformation, number of transformation foci per dish. Cells were cotreated with 10 mM NAC and the metal mixture (2 *μ*M NaAsO_2_, 2 *μ*M CdCl_2_, and 5 *μ*M Pb(C_2_H_3_O_2_)_2_·3H_2_O); samples were collected on day 25. Data represent the mean of three independent experiments performed in triplicate. Transformation is presented as the fold change with respect to control values. This value corresponds to the number of foci/dish in the experimental condition over the number of foci/dish in control samples. The results are presented as a percentage with respect to control values. ANOVA and Student's *t*-test *P < 0.05, **P < 0.001, ***P < 0.0001.

Transformation was determined by measuring the foci number per dish on day 25 (Figure 7). NAC cotreatment diminished transformation when is administered as initiator, whereas NAC cotreatment abolished metal-mixture-induced transformation as promoter treatment. NAC cotreatment as initiator and promoter had an intermediate effect; it diminished the number of transformation foci (Figure 7). These results suggest that oxidative stress greatly influences the promotion phase but not the initiation phase; thus, a non-oxidation-stress mechanism must underlie the effects observed during the initiation phase.

## 4. Discussion

Numerous metals found in the environment have been classified as carcinogens [7–9]. Acute exposure to these metals is common; however, in smelter and recycling battery industries, people are exposed chronically to metal mixtures of NaAsO_2_, CdCl_2_, and Pb(C_2_H_3_O_2_)_2_·3H_2_O, having this kind of exposure as our particular interest [1, 23–25]. Several studies have been performed to find an interaction profile of this mixture suggesting an additive interaction in carcinogenic process [5, 6]. *In vitro* cell transformation assays have shown a relatively high correlation to carcinogenicity bioassays; one of the most valuated models are Balb/c 3T3 cells, in which we can evaluate both initiator and promoter substances in transformation process [26]. Because cell transformation is an event that is related with this carcinogenic capacity, we evaluated whether a metal mixture of 2 *μ*M NaAsO_2_, 2 *μ*M CdCl_2_, and 5 *μ*M Pb(C_2_H_3_O_2_)_2_·3H_2_O could transform Balb/c 3T3 cells. These cells have recently been mentioned as good model to the study of mixtures [37]. We evaluated the effects of the mixture as initiator, promoter, and both and determined the role of oxidative stress in transformation process. Our results showed that metals mixtures (2 *μ*M NaAsO_2_, 2 *μ*M CdCl_2_, and 5 *μ*M Pb(C_2_H_3_O_2_)_2_·3H_2_O) produced transformation as both initiator and promoter (Tables 1 and 2), since other studies showed that single exposure to these metals could not produced transformation at the same concentrations except for cadmium. It appears that mixture enhances morphological transformation in this model [16, 21, 22, 38]. Meanwhile, concerning transforming potential, which is a measure of the capacity of the cells to produce foci, we detect a strong effect when metal mixture was administered as promoter more than initiator, pointing out the relevance of this particular mixture in the carcinogenic process. Cadmium, on the other hand, has been proposed as promoter in the model by several authors [21, 25, 26, 34, 39–42]. This is in agreement with the idea that metal carcinogenic could be more related with promotion effects rather than initiation effects.

**Figure 8:**
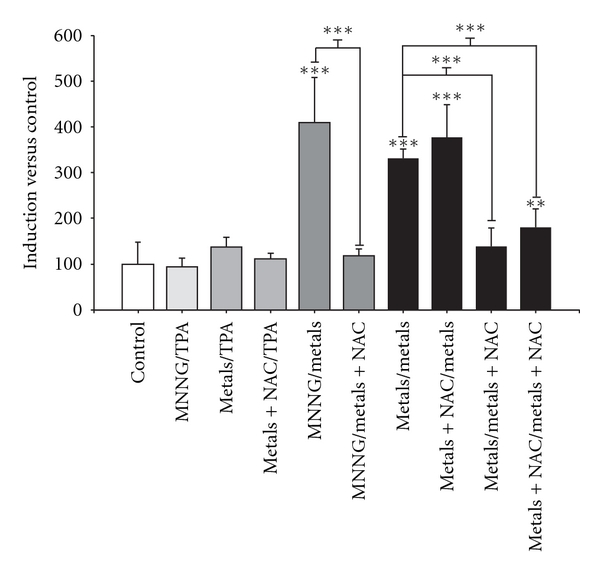
Influence of *N*-acetyl-cysteine (NAC) on metal-mixture-induced transformation, lipid peroxidation. Cells were cotreated with 10 mM NAC and the metal mixture (2 *μ*M NaAsO_2_, 2 *μ*M CdCl_2_, and 5 *μ*M Pb(C_2_H_3_O_2_)_2_·3H_2_O); samples were collected on day 16. Data represent the mean of three independent experiments performed in triplicate. LPx was measured as an oxidative stress marker. The results are presented as a percentage with respect to control values, as determined by the thiobarbituric acid method (ANOVA and Student's *t* test **P < 0.001, ***P < 0.0001).

**Figure 9:**
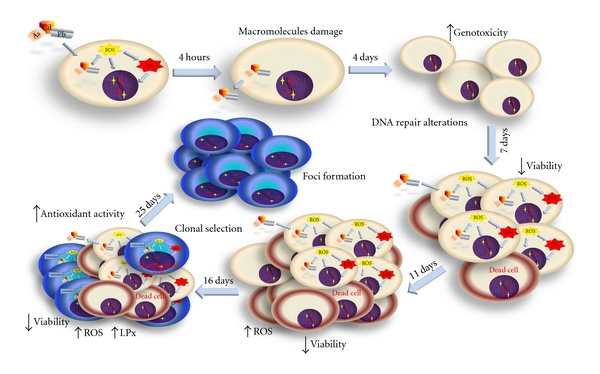
Scheme of metal mixture transformation process in Balb c/3T3. We show that damage to macromolecules (lipids and DNA) occurs the first day of initiation phase with no change in cell viability, suggesting that DNA repair systems may have been impaired by the metal mixture treatment (2 *μ*M NaAsO_2_, 2 *μ*M CdCl_2_, and 5 *μ*M Pb(C_2_H_3_O_2_)_2_·3H_2_O), in addition to the observed genotoxicity. During the promotion phase, clonal selection of the transformed cells was clearly observed, as evidenced by an increase in oxidative stress markers, induction of the antioxidant response, and loss of cell viability. Cells with various advantages survived, such as those with a high antioxidant capacity until foci formation.

The primary variables that affected transformation were the generation of reactive oxygen species (ROS) and the loss of cell viability; these data support the clone selection theory [10] (Tables 3 and 4, Figures 6 and 7). We statistically analyzed the contribution of ROS, lipoperoxidation, genotoxicity, SOD activity, catalase activity, TAC, and viability with the transforming potential across initiation and promotion phases. However, only ROS generation and loss of cell viability correlated significantly with transformation (Tables 3 and 4). We summarize our observations of the phenomenon in Figure 9; we show that oxidative damage to macromolecules is induced during the initiation phase (Figure 2) with no change in cell viability (Table 1). Considering that DNA damage occurred during the first days of the initiation process (Figure 2), we suggest that the DNA repair system may have been impaired by the metal mixture treatment, in addition to the observed genotoxicity; these effects have been observed in other studies as well [43–46]. During the promotion phase, clonal selection of the transformed cells was clearly observed, as evidenced by an increase in oxidative stress markers (ROS generation), induction of the antioxidant response, and loss of cell viability on day 16 (Figures 2–5, Table 2). Cells with various advantages survived, such as those with a high antioxidant capacity (Figure 5). These data agree with the results reported by Salnikow et al. [47], who determined that nickel-transformed Balb/c 3T3 cells demonstrate a high antioxidant capacity compared to nontransformed Balb/c 3T3 cells [47]. In addition, tumor cells possess an elevated antioxidant capacity [48, 49]. The present results suggest that oxidative stress is involved in metal-mixture-induced transformation, because elevated levels of oxidative markers were observed during both phases, and these effects induced clone selection of the transformed cells. To confirm these results, cotreatment of metal mixture (2 *μ*M NaAsO_2_, 2 *μ*M CdCl_2_ and 5 *μ*M Pb(C_2_H_3_O_2_)_2_·3H_2_O) with NAC was performed (Figures 6–9). This treatment abolished the observed transforming effects when it was performed as promoter treatment. However, the effect of the treatment was diminished when it was performed as initiator stimuli or both. These results suggest that a different mechanism may underlie the effects of the metal mixture during the initiation of transformation, and this mechanism may involve DNA damage, DNA repair alterations, or the regulation of gene expression. Nevertheless, during the promotion phase, the induction of oxidative stress plays an important and definitive role in metal-mixture-induced cell transformation. Our results suggest that oxidative stress induced by the metal mixture (2 *μ*M NaAsO_2_, 2 *μ*M CdCl_2_ and 5 *μ*M Pb(C_2_H_3_O_2_)_2_·3H_2_O) in Balb/c 3T3 cells leads to clone selection.

## Figures and Tables

**Table 1:** Transforming potential through initiation phase.

Initiation phase					
Day	Treatment^a^	Viability (%)^b^	Dish with foci/dish scored^c^	No. of transformed foci/dish	TP^d^
1	Control	100 ± 5.5	4/6	1.8 ± 0.05	1 ± 0.27
	MNNG + TPA	96 ± 4.8	6/6	62.25 ± 9.9***	35.37 ± 5.6***
	Metal mixture + TPA	100 ± 1.7	6/6	33.75 ± 11.6***	18.41 ± 6.3***
	MNNG + metal mixture	96 ± 4.8	6/6	23 ± 7.7***	2.61 ± 0.88
	Metal mixture + metal mixture	100 ± 1.7	6/6	29.5 ± 5.7***	3.22 ± 0.62
4	Control	100 ± 0.0	4/6	1.8 ± 0.05	1 ± 0.27
	MNNG + TPA	89 ± 5.8	6/6	62.25 ± 9.9***	22.7 ± 3.6***
	Metal mixture + TPA	89 ± 9.1	6/6	33.75 ± 11.6***	12.4 ± 4.2***
	MNNG + metal mixture	89 ± 5.8	6/6	23 ± 7.7***	3.2 ± 1.1
	Metal mixture + metal mixture	89 ± 9.1	6/6	29.5 ± 5.7***	1.9 ± 0.4
7	Control	100 ± 0.25	4/6	1.8 ± 0.05	1 ± 0.27
	MNNG + TPA	83 ± 2.8**	6/6	62.25 ± 9.9***	83.5 ± 13.4***
	Metal mixture + TPA	93 ± 0.12**	6/6	33.75 ± 11.6***	66.5 ± 22.8***
	MNNG + metal mixture	83 ± 2.8**	6/6	23 ± 7.7***	6.2 ± 2.1*
	Metal mixture + metal mixture	93 ± 0.12**	6/6	29.5 ± 5.7***	9.4 ± 1.8*

^a ^Treatments were added at concentrations of MNNG 0.5 *μ*g/mL, TPA 0.1 *μ*g/mL, and metal mixture (As 2 *μ*M, Cd 2 *μ*M, and Pb 5 *μ*M); ^b^percentage with respect to controls; ^c ^two experiments with three biological replicates; ^d^transforming potential (TP) calculated as number of transformed foci type III per dish/surviving cells at corresponding sampling day. Statistical analysis, Student's *t*-test *P* value *< 0.05, **< 0.01, and ***< 0.001.

**Table 2:** Transforming potential through promotion phase.

	Promotion Phase				
Day	Treatment^a^	Viability (%)^b^	Dish with foci/dish scored^c^	No. of transformed foci/dish	TP^d^
11	Control	100 ± 0.5	4/6	1.8 ± 0.05	1 ± 0.27
	MNNG + TPA	77 ± 19.1*	6/6	62.25 ± 9.9***	61.6 ± 9.8***
	Metal mixture + TPA	57 ± 15.8*	6/6	33.75 ± 11.6***	62 ± 21.3***
	MNNG + Meta mixture	9 ± 0.66***	6/6	23 ± 7.7***	70.5 ± 23.8***
	Metal mixture + metal mixture	17 ± 0.3***	6/6	29.5 ± 5.7***	78.5 ± 15.1***
16	Control	100 ± 0.0	4/6	1.8 ± 0.05	1 ± 0.27
	MNNG + TPA	100 ± 11.5	6/6	62.25 ± 9.9***	67.9 ± 10.8***
	Metal mixture + TPA	100 ± 18.5	6/6	33.75 ± 11.6***	25.1 ± 8.6***
	MNNG + metal mixture	31 ± 8.9**	6/6	23 ± 7.7***	38.5 ± 13***
	Metal mixture + metal mixture	30 ± 8***	6/6	29.5 ± 5.7***	55.2 ± 10.6***
21	Control	100 ± 0.0	4/6	1.8 ± 0.05	1 ± 0.27
	MNNG + TPA	77 ± 0.5***	6/6	62.25 ± 9.9***	42.6 ± 6.8***
	Metal mixture + TPA	88 ± 2.2**	6/6	33.75 ± 11.6***	11.7 ± 4*
	MNNG + metal mixture	76 ± 0.05***	6/6	23 ± 7.7***	13.6 ± 4.6*
	Metal mixture + metal mixture	31 ± 6.9***	6/6	29.5 ± 5.7***	50.5 ± 9.7***

^a^Treatments were added at concentrations of MNNG 0.5 *μ*g/mL, TPA 0.1 *μ*g/mL, and metal mixture (As 2 *μ*M, Cd 2 *μ*M, and Pb 5 *μ*M); ^b^percentage with respect to controls; ^c^two experiments with three biological replicates; ^d^transforming potential (TP) calculated as nmber of transformed foci type III per dish/surviving cells at corresponding sampling day. Statistical analysis, Student's *t*-test *P* value *< 0.05, **< 0.01 and ***< 0.001.

**Table 3:** Pearson's correlation between oxidative and antioxidant markers that influence transforming potential (TP).

Pearson's correlation Correlation coefficient (*r*)	ROS	LPx	Genotoxicity	SOD activity	Catalase activity	TAC	Viability
TP^a^	0.436*	0.071	−0.046	0.216	0.219	0.155	−0.753**
ROS^b^		0.730**	0.209	0.754**	0.881***	0.835***	−0.539*
LPx^c^			0.453*	0.909***	0.833***	0.873***	−0.282
Genotoxicity				0.582*	0.445*	0.149	−0.001
SOD^d^ activity					0.848***	0.805***	−0.439*
Catalase activity						0.782***	−0.429
TAC^e^							−0.398

Values represent correlation coefficient (*r*) and *P* values *P < 0.05, **P < 0.001, ***P < 0.0001. ^a^TP: transforming potential; ^b^ROS: reactive oxygen species; ^c^LPx: lipid peroxidation; ^d^SOD: superoxide dismutase; ^e^TAC: total antioxidant capacity.

**Table 4:** Multivariate analysis of markers related with transforming potential (TP).

Multiple linear regression	Coefficient	Standard error	*P* value	VIF^a^
ROS^b^	0.183	0.076	0.037	8.545*
LPx^c^	0.023	0.034	0.506	12.405
Genotoxicity	−0.006	0.096	0.948	3.346
SOD^d^ activity	0.008	0.096	0.934	12.940
Catalase activity	−0.112	0.068	0.132	8.732
TAC^e^	−0.028	0.018	0.149	10.827
Viability	−0.373	0.123	0.013	1.926*

^a^VIF: variance inflation factor; ^b^ROS: reactive oxygen species; ^c^LPx: lipid peroxidation; ^d^SOD: superoxide dismutase; ^e^TAC: total antioxidant capacity. *P < 0.05.
